# Helping Adolescents with Greater Psychosocial Needs: Subjective Outcome Evaluation Based on Different Cohorts

**DOI:** 10.1100/2012/694018

**Published:** 2012-04-01

**Authors:** Daniel T. L. Shek, Tak Yan Lee

**Affiliations:** ^1^Department of Applied Social Sciences, The Hong Kong Polytechnic University, Hong Kong; ^2^Public Policy Research Institute, The Hong Kong Polytechnic University, Hong Kong; ^3^Department of Social Work, East China Normal University, Shanghai, China; ^4^Kiang Wu Nursing College of Macau, Macau; ^5^Division of Adolescent Medicine, Department of Pediatrics, Kentucky Children's Hospital, University of Kentucky College of Medicine, Lexington, KY, USA; ^6^Department of Applied Social Studies, City University of Hong Kong, Hong Kong

## Abstract

The Tier 2 Program of the Project P.A.T.H.S. (Positive Adolescent Training through Holistic Social Programmes) is designed to help students with greater psychosocial needs. This paper examines nine sets of subjective outcome evaluation data collected from 2005 to 2009 (*n* = 60, 241 participants). Based on the consolidated data with schools as units, results showed that participants generally had positive perceptions of the program, implementers, and benefits of the program. The subjective outcome evaluation instrument was found to be internally consistent. Multiple regression analysis revealed that perceived qualities of the program and the program implementers predicted perceived effectiveness of the program. The present study provides support for the effectiveness of the Tier 2 Program of the Project P.A.T.H.S. in Hong Kong.

## 1. Introduction

The Project “P.A.T.H.S. to Adulthood: A Jockey Club Youth Enhancement Scheme” (“P.A.T.H.S.” denotes Positive Adolescent Training through Holistic Social Programmes) is a large-scale positive youth development program designed for junior secondary school students (Secondary 1 to 3, equivalent to Grades 7 to 9 in the North American system) in Hong Kong [1, 2]. There are two tiers of programs (Tier 1 and Tier 2 Programs) in this project [3]. The Tier 1 Program is a universal prevention initiative in which Secondary 1 to 3 students take part. Using a structured curriculum, either a 20 h full program or a 10 h core program is provided each school year for each grade. Students learn competencies with reference to the 15 positive youth development constructs as identified in the successful programs by Catalano et al. [4]. Based on different evaluation strategies, including objective outcome evaluation, subjective outcome evaluation, process evaluation, qualitative evaluation, and interim evaluation, there is evidence supporting the effectiveness of the Tier 1 Program [5–7].

Besides building up psychosocial competencies in adolescents via the Tier 1 Program, students with greater psychosocial needs are helped via the Tier 2 Program. Because research findings suggest that roughly one-fifth of adolescents need more help, the Tier 2 Program is provided for at least one-fifth of the students who display greater psychosocial needs at each grade (i.e., selective prevention) by the school social work service providers. The researchers deliberately avoid using the term “at risk” because the term is very stigmatizing in the Chinese culture, and it deters parents and students from joining the related programs. The Tier 2 Program (Selective Program) targets adolescents with greater psychosocial needs who are identified in the Tier 1 Program and/or via other sources. Students with greater psychosocial needs usually have special needs in the academic, personal (e.g., adjustment, mental health, and values concerns), interpersonal, and familial domains. Information based on multiple sources, including objective assessment tools (e.g., Family Assessment Instrument, Life Satisfaction Scale, Hong Kong Student Information Form), teachers' ratings, student records, and other relevant quantitative and qualitative information based on systematic assessment, is used to identify students for the Tier 2 program.

Services based on the Tier 2 Program were provided for students who were identified as having special needs. Some schools also involved the parents of these students. To create more flexibility for the workers with reference to the specific needs of the students in the unique school context, the applicants (i.e., the school social work service agencies) had the choice of designing appropriate programs that target the needs of the students with reference to the 15 positive youth development constructs maintained in the project, as well as goals and objectives covered in this project [8]. Several nonmutually exclusive examples for the Tier 2 Program include (1) mentorship programs involving the alumni of the schools, (2) mental health promotion programs, (3) adventure-based counseling, (4) parenting programs, (5) service learning programs, and (6) resilience enhancement programs.

In a pioneering study of the subjective outcome evaluation of the Tier 2 Program (Secondary 1 level) students in the Experimental Implementation Phase carried out in 2005/2006, the first author and colleagues [9] conducted a subjective outcome evaluation on 2,173 participants from 52 schools after they completed the Tier 2 Program. Based on the consolidated reports submitted by the agencies to the funding body, the research team aggregated the consolidated data to form a “reconstructed” overall profile on the perceptions of the program participants. Four major types of program were identified, including programs based on the adventure-based counseling (ABC) approach, programs concentrated on volunteer training and services (VTSs), programs incorporating both ABC and VTS elements, and other programs with different foci. Results showed that high proportions of the respondents had positive perceptions of the programs and the instructors, and roughly four-fifths of the respondents regarded the program as helpful to them. However, it is noteworthy that there is no specific relationship between the types of Tier 2 Programs and psychosocial needs of the participants.

In the first year of the Full Implementation Phase (2006/2007), 10,255 participants (Secondary 1 level) from 207 schools joined the Tier 2 Program. Shek and Sun [10] conducted a similar study and found that the same high proportions of the respondents had positive perceptions of the programs and the implementers, and around four-fifths of the respondents regarded the program as helpful to them. Three subsequent studies also showed the effectiveness of the Tier 2 Program in the holistic development of the program participants. These included a study on 1,898 Secondary 2 level participants from 49 schools in the second year of the Experimental Implementation Phase in 2006/2007 [11], a replication study conducted by Lee and Shek [12] on 9,931 participants from 212 schools (Secondary 1 level) in the second year of the Full Implementation Phase in 2007/2008, and a study on 1,739 Secondary 3 level participants from 48 schools in the third year of the Experimental Implementation Phase in 2007/2008 [13]. Very similar results were found from these five studies showing that roughly the same high proportions of the respondents had positive perceptions of the programs and the instructors, and over four-fifths of the respondents regarded the program as helpful to them. These subjective outcome evaluation studies provide evidence supporting the effectiveness of the Tier 2 Program at the Secondary 1 to 3 levels in the Experimental Implementation Phase and at the Secondary 1 level in the Full Implementation Phase.

The quality of the program and the workers implementing the program are important determinants of program effectiveness. Nation et al. [14] pointed out that a comprehensive program and well-trained program implementers are important elements of an effective program. Weissberg [15] also pointed out that a well-designed program and high-quality program implementers were commonly found in effective school-based social-emotional learning programs. There are also research findings showing that qualities of the program implementation and program implementers are related to program outcomes. For example, Harachi et al. [16] showed that instructional strategies (proactive classroom management, cooperative learning methods, strategies to enhance student motivation, student involvement and participation, reading strategies, and interpersonal and problem-solving skills training) were related to student social competencies. Tobler et al. [17] also showed that programs with high peer interaction were more effective than programs with low peer interaction and that the delivery method instead of the program content determined the success of the program. According to these research findings, it was hypothesized that positive perceptions on program characteristics and program implementers were predictors of perceived helpfulness of the program.

Regarding the different modes of Tier 2 Programs, although previous studies showed that ABC and VTS were the two major modes involved [9–13], comparative analyses were not meaningful because of the variations in the unique design of programs for different schools. As far as ABC is concerned, it is an approach that attempts to integrate adventure, wilderness, and experiential learning, as well as individual and group counseling [18–21]. According to this approach, when an adolescent with a disequilibrium in personal development is put into an environment that is strange and requires cooperation (i.e., adventure environment), the tasks designed to provide adventure experiences will lead to transformation in the participant, including changes in self-confidence and self-understanding, as well as cooperation with others [21–25]. According to Schoel et al. [26], ABC promotes life skills in the participants, including communication skills, cooperation, and decision-making and problem-solving skills. With regard to the effectiveness of ABC, Moote and Wodarski showed that 16 of the 19 studies under review reported some positive effects for the participants, including enhanced self-esteem, self-concept, cooperative behavior, and physical, social, and intellectual growth [27]. They also concluded that “for social workers who provide direct services to adolescents in various settings, adventure-based counseling may be a viable alternative to traditional approaches” (pp. 161-162).

The second major mode of Tier 2 Programs is closely related to VTS. According to Clary et al. [28], there are six functions of volunteering. They are (1) enhanced understanding of the world through volunteering (knowledge function); (2) expression of values via volunteering (value expressive function); (3) avoidance of personal issues or undesirable truths about the self via volunteering (ego defensive function); (4) enhancement of self-esteem, competence, and mood (self-enhancement function); (5) facilitation of career and development of a better resumé (utilitarian function); (6) social companionship and socializing with other volunteers (interpersonal function). Clearly, research findings showed that volunteers perceived several benefits of volunteering, including knowledge and skill acquisition, enhancement in occupational and educational opportunities, and social belongingness among peers [29–32]. Other benefits for adolescents engaging in volunteerism include a lower level of anticipated distress and negative emotion [33, 34]. Based on these findings, it would be expected that VTS would promote positive youth development.

This paper presents and discusses the findings of a multicohort subjective outcome evaluation of the Tier 2 Program implemented in the Experimental and Full Implementation Phases of the P.A.T.H.S. Project. A total of nine cohort studies were implemented between 2005 and 2009 based on the perspective of the participants. Besides describing the profile of responses based on subjective outcome evaluation, the relationships between perceived quality of the program and program implementers and perceived program effectiveness were examined. Furthermore, whether participants who joined different programs differed in their evaluation of the program would be examined. It is noteworthy that although it is very common for social work agencies to design programs for adolescents with greater psychosocial needs (e.g., ABC, VTS), systematic evaluation and documentation of program evaluation have been rarely found in the local social work literature [35].

## 2. Methods

### 2.1. Participants and Procedures

From 2005 to 2009, the total number of schools that participated in the Project P.A.T.H.S. was 244. Since each school joined the Project for 3 or more years, the total number of “schools” as units of analyses was larger than 244. There were 669 schools in the Secondary 1 level, 443 in the Secondary 2 level, and 215 in the Secondary 3 level. Among them, 46.27% of the respondent schools adopted the full program (i.e., 20-h program involving 40 units), whereas 53.73% of the respondent schools adopted the core program (i.e., 10-h program involving 20 units). Altogether, 93,001 participants (48,212 at the Secondary 1 level, 29,644 at the Secondary 2 level, and 15,145 at the Secondary 3 level) joined the Tier 2 Program. Among these participants, 83,378 were student participants identified by teachers, parents, and/or self-administered questionnaires as having greater psychosocial needs, and 9,623 were their parents and teachers. The mean number of participants per school was 65.62 (range: 3–1,272). The mean number of sessions used for implementing the program was 22.36 (range: 1–120). The basic characteristics of the participants in the different datasets can be seen in Table 1.

After completing the Tier 2 Program, the participants were invited to respond to a Subjective Outcome Evaluation Form (Form C) developed by the first author [2]. From 2005 to 2009, a total of 60,241 questionnaires were completed (mean = 43.74 participants per school, range: 3–294) with an overall response rate of 64.78%. To facilitate the program evaluation, the research team developed an evaluation manual with standardized instructions for collecting the subjective outcome evaluation data [2, 36]. In addition, adequate training was provided to the implementers during the 20-h training workshops on how to collect and analyze the data collected by Form C.

On the day when the evaluation data were collected, the purpose of the evaluation was mentioned, and the confidentiality of the data collected was repeatedly emphasized to all of the respondents. The respondents were asked to indicate if they did not want to respond to the evaluation questionnaire (i.e., “passive” informed consent was obtained). All respondents responded to all scales in the evaluation form in a self-administration format. Adequate time was provided for the respondents to complete the questionnaire.

### 2.2. Instruments

The Subjective Outcome Evaluation Form (Form C) [36] was used to measure the participants' perceptions of Tier 2 Program. There are seven parts in this evaluation form:

- participants' perceptions of the program, such as program design, quality of service, appropriateness of the program, and interaction among the participants (8 items),
- participants' perceptions of the workers, such as the preparation of the workers, professional attitude and knowledge, and interaction with the participants (8 items),
- participants' perception of the effectiveness of the program, such as promotion of different psychosocial competencies, resilience, and overall personal development (8 items),
- things that the participants appreciated most (open-ended question),
- opinion about the workers (open-ended question),
- things that the participants learned from the program (open-ended question),
- areas that require improvement (open-ended question).

For the quantitative data, the implementers collecting the data were requested to input the data into an EXCEL file developed by the research team that would automatically compute the frequencies and percentages associated with the different ratings for an item. When the schools submitted the reports, they were also requested to submit the soft copy of the consolidated datasheets. After receiving the consolidated data by the funding body, the data were aggregated in order to “reconstruct” the overall profile based on the subjective outcome evaluation data by the research team. Only quantitative data based on the rating scale items were examined in this study.

## 3. Data Analyses

Percentage findings were examined using descriptive statistics. A composite measure of each factor (i.e., perceived qualities of program content, perceived qualities of program implementers, and perceived program effectiveness) was created based on the total scores of each factor divided by the number of items. Pearson's correlation analyses were performed to examine if the program content and program implementers were related to the program effectiveness. Multiple regression analyses were performed to test if and how well each factor would predict the program effectiveness. All analyses were performed by using the Statistical Package for Social Sciences Version 17.0.

## 4. Results

The characteristics of the Tier 2 Programs implemented from the 2005/2006 to the 2008/2009 school year can be found in Table 2, which included the number of participants, program attendance, number of program aims and constructs, as well as the mean overall effectiveness. The program participants involved students, parents, and teachers. Among the four program approaches, Type A (ABC plus VTS) was the most widely employed approach, which was used in 525 out of 1,326 programs. This was followed by Type B (ABC only), which accounted for 373 programs, and then Types C (VTS only, 220 programs) and D (approaches other than ABC or VTS, 208 programs). The average number of participants ranged from 50.58 to 71.18, with the average program attendance ranging from 81.15 to 86.06%. The mean overall effectiveness of all Tier 2 Programs ranged from 4.49 to 4.76 on a 6-point Likert scale toward the positive side.

The quantitative findings based on the closed-ended questions are presented in this paper. Several observations can be highlighted from the findings. First, the participants generally had positive perceptions of the program (Table 3). On the whole, they were satisfied with the program (89.05%), they had much interaction with other participants (87.88%), and they perceived the service delivered could achieve the planned objectives (87.81%). Second, a high proportion of the participants had a positive evaluation of the program implementers (Table 4). For example, participants perceived that the workers had prepared well for the program (91.47%), had professional knowledge (90.82%), and had good attitudes (90.77%). Third, as shown in Table 5, participants had positive views toward the effectiveness of the program. The service enhanced participants' self-help skills (88.26%), problem-solving skills (87.73%), and growth (87.30%).

Reliability analysis with the schools as the unit of analyses showed that Form C was internally consistent (Table 6) including 8 items related to the program content (*α* = 0.99 for Secondary 1 through 3 participants), 8 items related to the implementer (*α* = 0.99 for Secondary 1 and 3 participants; *α* = 0.98 for Secondary 2 participants), 8 items related to the perceived benefits (*α* = 0.99 for Secondary 1 through 3 participants), and the 24 items measuring overall program effectiveness (*α* = 0.99 for Secondary 1 through 3 participants). Results of correlation analyses showed that both program content (*r* = 0.92, *P* < 0.01) and program implementers (*r* = 0.89, *P* < 0.01) were strongly associated with program effectiveness (Table 7).

Table 8 presents multiple regression analyses results. First, higher positive views toward the program content and program implementers were associated with higher program effectiveness (*P* < 0.01). Second, perception of program content was a stronger predictor for program effectiveness (*β* ranged from 0.59 to 0.65) than perception of program implementers (*β* ranged from 0.36 to 0.41). Third, the models explained a large amount of the variance toward the prediction of program effectiveness across grade levels.

Regarding possible differences among the different types of programs on different measures of subjective outcome evaluation, results of MANOVA did not show any significant difference among the four types of programs on participants' views on the program, their views on program instructors, as well as their perceived effectiveness of the program. The mean scores of the key variables for the four different types of programs can be seen in Table 9.

## 5. Discussion

The current study examined the Tier 2 Program of the Project P.A.T.H.S. in terms of participants' subjective perceptions of the program, the instructor, and effectiveness of the program based on nine datasets. There are several unique characteristics of the study. First, a large sample size was used. Second, several datasets collected over time were utilized. Third, a reliable measure of subjective outcome evaluation was employed to carry out subjective outcome evaluation. Fourth, this is the first known scientific study utilizing such a large sample to examine the perceived effectiveness of youth development programs developed for adolescents with greater psychosocial needs. Differences across cohorts were not specifically focused upon because there was nonindependence of observations across time.

Several observations can be highlighted from the present study. First, results showed that the various measures derived from Form C were internally consistent. With reference to the comments of Royse [37] that the lack of standardized assessment tools for conducting client satisfaction surveys also introduces biases for the client satisfaction approach and that the use of assessment tools with known reliability and validity would “eliminate many of the problems found in hastily designed questionnaires” (p. 265), the present study is an interesting addition to the literature. It is noteworthy that there are very few validated subjective outcome evaluation measures in different Chinese contexts.

Second, high proportions of the participants perceived the program in a positive light. Most of the respondents had favorable perceptions of the program and workers implementing the program; roughly four-fifths of the respondents perceived the program to be beneficial to their own development. These observations generally suggest that the Tier 2 Program was perceived positively by the program participants and they felt that the program was helpful to them. These findings are also consistent with those findings emerging from the separate analyses in the Experimental and Full Implementation Phases suggesting that the Tier 2 Program is beneficial to the program participants. Of course, these positive findings should be viewed together with the limitations of subjective outcome evaluation, such as demand characteristics and halo effect. If resources permit, studies attempting to examine the convergence of objective outcome evaluation findings and subjective outcome evaluation findings should be carried out.

Third, consistent with our expectations, participants' perceptions of the program were positively correlated with perceived benefits of the program. Similarly, participants' perceptions of the program implementers were positively correlated with perceived benefits of the program. These findings basically suggest that the quality of the program and program implementers are intimately related to perceived benefits of the program. Because there are few studies examining the correlates of program effectiveness based on a subjective outcome evaluation approach in the Chinese culture, the present findings can be regarded as pioneering additions to the literature [9–13, 38, 39].

The present findings give some insights into the differences in subjective outcome evaluation findings across different programs. The findings showed that there were no differences between the different types of programs in the Tier 2 Program. Although ABC has a long history in Hong Kong and it has been formally adopted as the major program theory for a huge social intervention program entitled “The Understanding the Adolescent Project” (UAP) to combat the problems among students identified as adolescent at risk from 2001 to 2004 in Hong Kong [40, 41], very few studies have systematically examined its effectiveness. Similarly, although volunteer programs are commonly used in Hong Kong, few systematic research studies have been carried out. Nevertheless, as the present findings were “reconstructed” from the evaluation reports submitted by the agencies, the units of analyses were schools instead of individuals. As such, the power of the statistical analyses would become low and individual variations were lost in the process. Obviously, analyses based on data collected from individual participants should be attempted in the future. Finally, it is noteworthy that the mean ratings for the different types of programs are all on the high side, suggesting that they were perceived equally positively by the program participants.

Finally, the design and implementation of the Tier 2 program largely accomplished the six general characteristics of effective prevention efforts proposed by Flannery [42]: (1) focus on enhancing protective factors and social competencies as well as on risk reduction, (2) base on scientific evidence (research-based) and implement with high quality and fidelity, (3) target multiple outcomes at multiple levels (e.g., combine school and family/community efforts), (4) be culturally sensitive and developmentally (age) appropriate, (5) help youth learn how to apply social-emotional skills and ethical values in daily life, and (6) encourage responsibility, connection to prosocial peers, attachment to institutions, and relationships with prosocial adult mentors, all of which can decrease the likelihood of risky behavior.

In the area of human services, subjective outcome evaluation is commonly used [43–45], and there is convergence of subjective outcome evaluation and objective outcome evaluation [46]. However, it is noteworthy that there are several limitations in the present study. The first limitation is a relatively low overall response rate (64.78%) on the Subjective Outcome Evaluation Form (Form C). There are three plausible reasons for the low response rate. First, participants withdrew from the Tier 2 Program before completion. Second, participants were absent from the last session and did not complete the evaluation form. Third, some schools did not invite the adult participants to respond to the evaluation form. Future studies are encouraged to notify and invite adult participants to complete the evaluation form, as well as to encourage participants to attend the last session of the program. The second limitation is that details on participant composition are not available. Although each program is categorized as having one of the four participant compositions (i.e., students only; students and parents; students and teachers; students, parents, and teachers), the exact number of each type of participant within individual programs is not available, making it impossible to examine whether different participant groups (such as parents or students) may have different views toward the program. The third limitation is that these nine cohort studies were all subjective outcome evaluation. We must be very careful in drawing conclusions about the effectiveness of the Tier 2 Program. However, it is noteworthy that there are research findings showing that there is a convergence of subjective outcome evaluation and objective outcome evaluation.

To sum up, the present study provided a general picture of the implementation and subjective outcome of the Tier 2 Program of the Project P.A.T.H.S. from the perspective of the participants based on a series of studies in the Experimental and Full Implementation Phases. The study evidenced the worth of the Tier 2 Program in that it is perceived positively by almost all the participants. In addition, both program content and program implementers were found to be predictors of perceived effectiveness of the program. Finally, program type was found to be unrelated to the different domains of subjective outcome evaluation findings. These findings have important implications for the design of positive youth development programs in the future. In conjunction with other evaluation findings [7, 47–50], the present study suggests that the Project P.A.T.H.S. can promote development of adolescents in Hong Kong.

## Figures and Tables

**Table 1:** Description of data characteristics from 2005 to 2009.

	S1				S2			S3	
	2005/2006 EIP	2006/2007 FIP	2007/2008 FIP	2008/2009 FIP	2006/2007 EIP	2007/2008 FIP	2008/2009 FIP	2007/2008 EIP	2008/2009 FIP
Total schools that joined P.A.T.H.S.	52	207	213*	197	49	196	198	48	167
(i) 10-h program	23	95	108	104	27	113	110	29	104
(ii) 20-h program	29	112	105	93	22	83	88	19	63
Tier 2 Program	
Mean no. of sessions of program implementation	19.53 (1–63)	22.91 (6–62)	22.71 (8–120)	22.11 (5–76)	22.63 (1–62)	23.13 (5–119)	22.04 (5–77)	22.77 (10–55)	23.39 (5–90)
Hours per session	1.5–3	1.5–3	1.5–3	1.5–3	1.5–3	1.5–3	1.5–3	1.5–3	1.5–3
Total no. of participants	3,072	13,194	15,494	16,452	2,542	12,490	14,612	2,114	13,031
(i) Students	2,718	12,092	13,032	14,192	2,439	11,347	13,382	2,114	12,062
(ii) Adults	354	1,102	2,462	2,260	103	1,143	1,230	0	969
Mean no. of participants per school	59.08 (21–274)	63.74 (14–308)	72.74 (13–360)	83.51 (3–1272)	51.88 (17–240)	63.72 (10–435)	73.80 (15–351)	44.04 (6–93)	78.03 (9–406)
Total no. of respondents	2,173	10,255	9,931	9,216	1,898	8,485	9,166	1,739	7,378
Mean no. of student respondents per school	41.79 (20–151)	49.54 (6–294)	46.84 (7–198)	46.78 (3–215)	38.73 (8–199)	43.29 (7–196)	46.29 (7–281)	36.23 (2–136)	44.18 (5–222)

*Note*. S1: Secondary 1 level; S2: Secondary 2 level; S3: Secondary 3 level; EIP: Experimental Implementation Phase, FIP: Full Implementation Phase.

*In the 2007/2008 school year, only 212 schools submitted the Tier 2 evaluation reports.

**Table 2:** Summary of the characteristics and effectiveness of the Tier 2 program.

Main program approach	Clientele	Average no. of participants	Average program attendance (%)	Average no. of program aims indicated in the reports	Average no. of constructs indicated in the reports	Mean of overall effectiveness
ABC plus VTS (Type A) (*n* = 525)	S1 (*n* = 315)	60.40	83.20	2.05	6.52	4.58
	S2 (*n* = 151)	50.58	81.56	2.49	6.56	4.64
	S3 (*n* = 59)	55.34	81.15	2.39	6.53	4.76

ABC only (Type B) (*n* = 373)	S1 (*n* = 196)	59.92	82.64	2.14	6.25	4.54
	S2 (*n* = 109)	58.44	81.53	2.06	6.65	4.59
	S3 (*n* = 68)	54.51	82.97	2.26	5.96	4.70

VTS only (Type C) (*n* = 220)	S1 (*n* = 82)	60.29	82.22	2.41	6.54	4.56
	S2 (*n* = 99)	54.35	82.54	2.37	6.61	4.62
	S3 (*n* = 39)	65.28	82.90	2.21	6.90	4.67

Other approaches (Type D) (*n* = 208)	S1 (*n* = 75)	67.03	86.06	2.09	5.24	4.56
	S2 (*n* = 84)	71.18	81.21	1.98	6.21	4.49
	S3 (*n* = 49)	61.65	83.72	2.20	5.65	4.61

**Table 3:** Summary of the students' perception towards the program.

		Respondents with positive responses (Options 4–6)							
		S1		S2		S3		Overall	
		*n*	%	*n*	%	*n*	%	*n*	%
(1)	The activities were carefully planned.	26,585	84.61	16,890	86.84	8,074	88.99	51,549	86.81
(2)	The quality of the service was high.	26,260	83.60	16,867	86.80	8,051	88.83	51,178	86.41
(3)	The service provided could meet the participants' needs.	26,462	84.31	16,914	87.15	8,111	89.56	51,487	87.01
(4)	The service delivered could achieve the planned objectives.	26,861	85.59	17,028	87.76	8,155	90.09	52,044	87.81
(5)	I could get the service I wanted.	25,844	82.42	16,457	84.86	7,953	87.93	50,254	85.07
(6)	I had much interaction with other participants.	26,869	85.77	17,037	87.91	8,138	89.97	52,044	87.88
(7)	I would recommend others who have similar needs to participate in the program.	25,202	80.48	16,113	83.16	7,777	86.03	49,092	83.22
(8)	On the whole, I am satisfied with the service.	27,196	86.82	17,292	89.15	8,241	91.18	52,729	89.05

*Note. *All items are on a 6-point Likert scale with 1 = strongly disagree, 2 = disagree, 3 = slightly disagree, 4 = slightly agree, 5 = agree, 6 = strongly agree. Only respondents with positive responses (Options 4–6) are shown in the table.

**Table 4:** Summary of the students' perception towards the worker(s).

		Respondents with positive responses (Options 4–6)							
		S1		S2		S3		Overall	
		*n*	%	*n*	%	*n*	%	*n*	%
(1)	The worker(s) has professional knowledge.	27,850	88.73	17,656	90.84	8,411	92.90	53,917	90.82
(2)	The worker(s) demonstrated good working skills.	27,373	87.26	17,488	90.03	8,312	91.84	53,173	89.71
(3)	The worker(s) was well prepared for the program.	28,153	89.81	17,765	91.58	8,419	93.03	54,337	91.47
(4)	The worker(s) understood the needs of the participants.	27,259	87.02	17,302	89.15	8,301	91.83	52,862	89.33
(5)	The worker(s) cared about the participants.	27,655	88.29	17,553	90.45	8,372	92.57	53,580	90.44
(6)	The worker(s)' attitudes were very good.	27,733	88.57	17,580	90.66	8,421	93.09	53,734	90.77
(7)	The worker(s) had much interaction with me.	26,470	84.49	16,926	87.31	8,123	89.84	51,519	87.21
(8)	On the whole, I am satisfied with the worker(s).	28,113	89.63	17,736	91.34	8,438	93.35	54,287	91.44

*Note. *All items are on a 6-point Likert scale with 1 = strongly disagree, 2 = disagree, 3 = slightly disagree, 4 = slightly agree, 5 = agree, 6 = strongly agree. Only respondents with positive responses (Options 4–6) are shown in the table.

**Table 5:** Summary of the students' perception towards the program effectiveness.

		Respondents with positive responses (Options 4–6)							
		S1		S2		S3		Overall	
		*n*	%	*n*	%	*n*	%	*n*	%
(1)	The service has helped me a lot.	25,968	83.45	16,405	85.40	7,907	88.01	50,280	85.62
(2)	The service has enhanced my growth.	26,513	85.27	16,663	86.79	8,070	89.85	51,246	87.30
(3)	In the future, I would receive similar service(s) if needed.	25,543	82.27	16,150	84.21	7,853	87.54	49,546	84.67
(4)	I have learned how to help myself through participating in the program.	26,881	86.62	16,858	87.98	8,090	90.18	51,829	88.26
(5)	I have positive change(s) after joining the program.	26,481	85.44	16,617	86.70	8,003	89.28	51,101	87.14
(6)	I have learned how to solve my problems through participating in the program.	26,618	86.20	16,699	87.50	7,996	89.50	51,313	87.73
(7)	My behavior has become better after joining this program.	25,172	81.17	15,792	82.40	7,635	85.21	48,599	82.93
(8)	Those who know me agree that this program has induced positive changes in me.	24,868	80.19	15,702	82.08	7,662	85.52	48,232	82.60

*Note. *All items are on a 6-point Likert scale with 1 = strongly disagree, 2 = disagree, 3 = slightly disagree, 4 = slightly agree, 5 = agree, 6 = strongly agree. Only respondents with positive responses (Options 4–6) are shown in the table.

**Table 6:** Means, standard deviations, Cronbach's alphas, and mean of interitem correlations among the variables by grade.

	S1		S2		S3		Overall	
	M (SD)	*α* (mean^#^)	M (SD)	*α* (mean^#^)	M (SD)	*α* (mean^#^)	M (SD)	*α* (mean^#^)
Program content (8 items)	4.58 (0.41)	0.99 (0.90)	4.64 (0.39)	0.99 (0.90)	4.73 (0.40)	0.99 (0.91)	4.62 (0.41)	0.99 (0.90)
Program implementers (8 items)	4.78 (0.39)	0.99(0.92)	4.84 (0.37)	0.98 (0.89)	4.93 (0.40)	0.99 (0.93)	4.83 (0.39)	0.99 (0.91)
Program effectiveness (8 items)	4.56 (0.41)	0.99(0.91)	4.59 (0.40)	0.99 (0.90)	4.69 (0.41)	0.99 (0.92)	4.59 (0.41)	0.99 (0.91)
Total effectiveness (24 items)	3.09 (0.26)	0.99 (0.86)	3.13 (0.25)	0.99 (0.84)	3.19 (0.26)	0.99 (0.88)	3.12 (0.26)	0.99 (0.86)

^#^Mean interitem correlations.

**Table 7:** Correlation coefficients among the variables.

Variable		1	2	3
(1)	Program content (8 items)	—		
(2)	Program implementers (8 items)	0.93**	—	
(3)	Program effectiveness (8 items)	0.92**	0.89**	—

**P < 0.01.

**Table 8:** Multiple regression analyses predicting program effectiveness.

	Predictors			
	Program content	Program implementer	Model	
	*β* ^ a^	*β* ^ a^	*R*	*R* ^2^
S1	0.60**	0.41**	0.99	0.98
S2	0.65**	0.36**	0.99	0.98
S3	0.59**	0.41**	0.99	0.99
Overall	0.61**	0.40**	0.99	0.98

^a^Standardized coefficients.

*P < 0.05.

**P < 0.01.

**Table 9:** Means and standard deviations among the variables by program type.

	ABC+VTS^1^	ABC^2^	VTS^3^	Others^4^	Overall
	M (SD)	M(SD)	M (SD)	M (SD)	M (SD)
Program content (8 items)	4.64 (0.41)	4.61 (0.39)	4.64 (0.41)	4.60 (0.41)	4.62 (0.41)
Program implementers (8 items)	4.83 (0.39)	4.80 (0.38)	4.85 (0.38)	4.81 (0.40)	4.83 (0.40)
Program effectiveness (8 items)	4.61 (0.42)	4.59 (0.39)	4.61 (0.42)	4.55 (0.41)	4.59 (0.41)
Total effectiveness (24 items)	3.13 (0.26)	3.11 (0.25)	3.13 (0.26)	3.10 (0.26)	3.12 (0.26)

^1^The program contents related to both ABC and VTS were indicated in the Tier 2 Program reports.

^2^The program contents related to ABC were indicated in the Tier 2 Program reports.

^3^The program contents related to VTS were indicated in the Tier 2 Program reports.

^4^Except ABC and VTS, other program contents were indicated in the Tier 2 Program reports.
